# Rates of Serious Surgical Errors in California and Plans to Prevent Recurrence

**DOI:** 10.1001/jamanetworkopen.2021.7058

**Published:** 2021-05-03

**Authors:** Andrew J. Cohen, Hansen Lui, Micha Zheng, Bhagat Cheema, German Patino, Michael A. Kohn, Anthony Enriquez, Benjamin N. Breyer

**Affiliations:** 1 James Buchanan Brady Urological Institute, Johns Hopkins Medical Institutions, Baltimore, Maryland; 2 Department of Urology, University of California, San Francisco; 3 Department of Biostatistics and Epidemiology, University of California, San Francisco

## Abstract

**Importance:**

Despite widespread recognition and known harms, serious surgical errors, known as surgical never events, endure. The California Department of Public Health (CDPH) has developed an oversight system to capture never events and a platform for process improvement that has not yet been critically appraised.

**Objectives:**

To examine surgical never events occurring in hospitals in California and summarize recommendations to prevent future events.

**Design, Setting, and Participants:**

This cross-sectional study identified 386 CDPH hospital administrative penalty reports, of which 142 were ascribable to never events occurring during surgery. These never events were identified and summarized from January 1, 2007, to December 31, 2017. A directed qualitative approach was used to analyze CDPH-mandated corrective steps to reduce future errors in this multicenter study of all accredited hospitals in California. Inclusion of surgical never event records was based on definitions established by the US Department of Health and Human Services National Quality Forum. Data analysis was performed from January 1, 2019, to November 30, 2020.

**Exposures:**

Never events include death or disability of an American Society of Anesthesiologists class I patient, wrong site or wrong surgery, retained foreign objects, burns, equipment failure leading to intraoperative injury, nonapproved experimental procedures, insufficient surgeon presence or privileges, or fall from the operating room table.

**Main Outcomes and Measures:**

Incident rates, consequences, and improvement plans to prevent additional never events were outcomes of interest.

**Results:**

A total of 142 never events were reported to the CDPH (1 per 200 000 operations). Annual surgical volume for hospitals with events was 9203 vs 3251 cases for hospitals without events (*P* < .001). A total of 94 of 142 events (66.2%) were retained foreign objects ranging from Kocher clamps to drain sponges. Wrong site or patient surgery accounted for 22 events (15.5%), surgical burns for 11 (7.7%), and other for 15 (10.6%). Other included insufficient surgeon presence, equipment failure, or falls in the operating room. Improvement plans included 18 unique categories of recommendations from regulators, many focusing on proper use of checklists. Regulators mandated a mean (SD) of 13 (7) corrective actions in the improvement plans. Policy adherence monitoring (119 [90.2%]), revision of existing policy (84 [63.6%]), and education regarding policy (83 [62.9%]) were common action items, whereas disciplinary action toward staff was rare (11 [8.3%]).

**Conclusions and Relevance:**

Surgical never events are a rare issue in California. Numerous strategies have evolved to reduce errors, many involving the thorough and proper use of intraoperative checklists.

## Key Points

**Question:**

What are the contemporary rates of serious surgical errors, and what are best practice changes to prevent further errors?

**Findings:**

This cross-sectional study identified 142 serious surgical errors from 2007 to 2017 in California hospitals. Subsequent surgery was required in 96 cases (67.6%), although death was rare. Regulators suggested 18 distinct strategies to prevent repeated events.

**Meaning:**

The findings suggest that surgical error remains a complex, rare issue in California driven by surgical volume; numerous strategies have evolved to prevent future events.

## Teaser

This cross-sectional study examines surgical never events occurring in hospitals in California and summarizes recommendations to prevent future events.

## Introduction

Among medical errors, surgical errors have the most direct and serious consequences, such as death or permanent injury.^1^ Serious surgical errors such as wrong-site surgery and retention of foreign objects (RFOs) are so-called never events that are potentially devastating in scope, cost, and impact on human health.^2^ Contemporary never events are commonly preventable and include RFOs and wrong-procedure, wrong-site, and wrong-patient surgery.^3^ The financial burden to hospitals is consequential, with reimbursement and institutional reputation negatively affected by these events.^4^ Moreover, approximately 75% of lawsuits against surgeons in the US are filed because of intraoperative error.^5^ The annual cost of medical errors likely exceeds $17 billion, with 35% being surgery related.^6^

Given widespread public recognition of the problem through publications such as *To Err is Human: Building a Safer Health System*,^7^ efforts to reduce error have been implemented into practice. Surgical checklists, for example, have been popularized as 1 method to prevent harm during surgery.^8,9,10^ Because of the complexity of medical systems or misuse, no checklist is 100% effective. Surgical never events persist on a multifaceted basis: a Joint Commission analysis revealed 1156 unique contributing causes, most frequently, human factors, lack of leadership, and poor communication.^11,12^ The study of surgical never events must first focus on understanding the scope of the issue and second address deficiencies in the environment that allow such events to take place.^13^ These steps may help fill in the gaps in which checklists fail.

California has a legally binding mechanism to record and publicize never events via the California Department of Public Health (CDPH) Center for Health Care Quality’s Licensing and Certification Program.^14^ Events are detected through unannounced inspections, public reporting, or hospital self-report. Such incidents are followed by an investigation by the CDPH. California is the most populous state in the US, with 39 million residents, and is well suited for the study of surgical errors.^15^ Although detailed surgical data have been recorded by the CDPH for more than a decade, to our knowledge, surgical never events have not been critically evaluated. Indeed, neither longitudinal studies of errors nor studies that outline concrete steps hospitals can and should take to prevent repeated events are featured in the literature.

Our objective was to describe the extent of reported surgical never events occurring in hospitals in California. Next, we analyzed hospital-level factors associated with rates of such errors. Finally, we used qualitative methods to summarize regulators’ suggestions to hospitals to prevent recurrent events. In other words, we identified concrete steps practitioners and administrators can take to prevent error based on best practice as viewed by regulators. In this way, we created a guide to help surgeons avoid never events, above and beyond the surgical checklist.

## Methods

In this cross-sectional study, data were collected from January 1, 2007, to December 31, 2017. Data analysis was performed from January 1, 2019, to November 30, 2020. These deidentified data are available in the public domain. The University of California, San Francisco Institutional Review Board denoted this research as exempt. This study followed the Standards for Reporting Qualitative Research (SRQR) reporting guideline.

### State Monitoring System

The CDPH Center for Health Care Quality’s Licensing and Certification Program monitors and tabulates hospital errors that are a concern to the safety of the public. When a facility violates requirements of licensure and negatively affects the health of patients, administrative penalties are issued as well as possible monetary fines. Periodic unannounced inspections of every licensed health facility occur at least once every 2 years. Complaints lodged by the public also result in inspections within 30 days. Furthermore, never events at each hospital must be self-reported, and an onsite inspection or investigation is required within 45 days.^14^

A CDPH team of at least 1 physician, 1 registered nurse, and 1 person experienced in hospital administration and sanitary inspections participates in detailed review of the event, including a site visit.^14^ If issues are confirmed, monetary penalties are $75 000 for the first deficiency, up to $100 000 for the second episode within 3 years, and up to $125 000 for the third and every subsequent violation.^14^ Penalties are also an opportunity for improvement: violations require a hospital to fulfill certain obligations as determined by the CDPH team to help ensure prevention of another incident. These requirements are listed in a detailed report. The facility is given 10 days to implement the improvement plans. Whether in person or through reviewing submitted data, the CDPH confirms hospitals followed through with said plan.

### Data Source

Data from all 386 publicly available CDPH hospital administrative penalty reports were accessed from January 1, 2007, to December 31, 2017.^14^ Such reports outline a wide spectrum of errors or incidents at hospitals. We defined a surgical never event based on definitions established by publication of the US Department of Health and Human Services National Quality Forum and considered only those that occurred in the operating room setting.^16^ For example, patients who experienced a medical error after surgery (such as in the recovery room, during an inpatient stay, or during an outpatient follow-up visit) were excluded. Never events include death or disability of an American Society of Anesthesiologists class I patient, wrong site or wrong surgery, RFOs, burns, equipment failure leading to intraoperative injury, non–institutional review board or US Food and Drug Administration experimental procedures, insufficient surgeon presence or privileges, or fall from the operating room table. All reports were reviewed by 2 authors (H.L. and M.Z.) for appropriateness for inclusion in the study. For cases in which consensus was not achieved, a third author (A.J.C.) provided arbitration. Additional data collected from each error report included hospital name, California Office of Statewide Health Planning and Development (OSHPD) hospital identification number, hospital location, the type of never event, subspecialty involved, and the consequence for the patient.

Consequences of never events were defined as the need for subsequent surgery, death, morbidity, or transfer of care. Transfer of care denotes a consequence in which a patient did not seek care at the same hospital; hence, no further outcome (such as repeat surgery performed elsewhere) was recorded. We transcribed the CDPH improvement plans for each penalty report.

Data regarding hospital and patient characteristics were obtained from the OSHPD, which publishes an annual report on licensed hospitals in California. The OSHPD mandates that each hospital in the state submit an annual utilization report. Data entry is performed by administrative staff yearly. Data herein include patient demographic information, such as age, race/ethnicity, insurance type, hospital zip code, discharge volume, number of procedures, number of hours of ambulance diversion by hospital stratified by year, trauma center designation, and teaching hospital status. Data from never event improvement plans and annual reports were matched using each hospital’s unique OSHPD identification number. To categorize hospitals based on whether they are situated in rural or urban areas, we used the rural-urban commuting area codes, which classify US Census tracts using measures of population density, urbanization, and daily commuting based on 2010 US Decennial Census data matched to zip codes.^17^ Metropolitan was defined as a rural-urban commuting area code less than 3.

### Qualitative Analysis

Qualitative analyses of improvement plans were performed using MAXQDA (Verbi Software) computer-assisted qualitative and mixed-methods analysis software. A directed qualitative content analysis approach was undertaken based on previously published qualitative research on hospital and surgical risk management.^18,19^ A coding system was adapted from the Systems Engineering Initiative for Patient Safety (SEIPS 2.0) model and the World Health Organization Surgical Checklist.^9,20,21^ The coding system was iteratively refined to include codes pertinent to this data set. The coding scheme was presented to a team of surgeons (A.J.C. and B.N.B.) who revised the codes to more effectively capture surgical practice. The final coding scheme consisted of broad categories (internal environment; interpersonal; organization; and tools, technology, and skills) that were subdivided into 55 subcodes (eAppendix 1 in the Supplement).

Events that met the inclusion criteria were summarized into a concise statement coded simultaneously by 2 different researchers (H.L. and M.Z.). To assess the intercoder reliability of the developed coding scheme, a subset of improvement plans were randomly selected and independently double coded. The 2 coders (H.L. and B.C.) then discussed outcomes from the analysis to reach a consensus and collaborated to refine the coding scheme. Intercoder reliability was assessed using the Cohen κ, and double coding ceased once the acceptable degree of intercoder agreement was reached (κ > 0.79).^22^ Analysis of the text was terminated on failure to generate additional inductive codes for each improvement plan. Descriptive analyses were performed to determine the coding frequencies of each plan of correction. A full accounting of the qualitative methods and details of adherence to the Standards for Reporting Qualitative Research are included in eAppendix 2 in the Supplement.

### Statistical Analysis

The penalty and hospital-level data were merged. Both χ^2^ and Fisher exact tests were performed to reveal differences among hospitals with never events vs not, with statistical significance set at *P* < .05. Hypothesis tests were 2-sided. The Kruskal-Wallis test applied for continuous variables. Given the rarity of events, Poisson regression models were also used to evaluate hospital characteristics associated with hospital penalties, accounting for multiple penalties in the same hospital and correcting for hospital volume. Included factors were determined a priori: teaching hospital status, metropolitan status, patient age, insurance mix, and race/ethnicity. Qualitative analysis proceeded as above, and simple descriptive statistics were applied to summarize findings. Additional analysis and graphics were created in Microsoft Excel (Microsoft Inc), Google Sheets (Alphabet Inc), and Stata (StataCorp).

## Results

### Number and Types of Never Events

From 2007 to 2017, we identified 386 penalty reports, of which 142 were ascribable to never events occurring during surgery (Table 1). Subsequent surgery was required in 96 cases (67.6%). Death was rare, occurring in 6 cases (4.2%). The RFOs were most common, accounting for 94 cases (66.2%). Among RFOs, retained sponges, gauze pads, or towels accounted for 57 of 94 cases (60.6%). The balance included assorted retained parts of a larger instrument (13 [13.8%]), clamps (8 [8.5%]), retractors (5 [5.3%]), wires (5 [5.3%]), or other (6 [6.4%]), such as bottles, drain bulbs, or cups. For RFOs, reexploration was performed 87.0% of the time. In 4 cases, the retained object was eliminated in feces, the object was not found during an exploration, or after discussion of risks and benefits it was decided to avoid reexploration entirely. For 90 reexplorations performed to address RFOs, 2 cases (2.2%) had complications because of the second operation itself, such as massive hemorrhage from the spleen (requiring splenectomy) and acute kidney failure.

**Table 1.** Type and Consequence of Never Events Stratified by Subspecialty

	No. (%) of never events				
	Wrong site, patient, or procedure (n = 22)	Unintended RFOs (n = 94)	Surgical burn (n = 11)	Other (n = 15)^a^	Total (N = 142)
Consequence					
Repeat surgery	10 (45.5)	82 (87.2)	4 (36.4)	0	96 (67.6)
Morbidity	11 (50.0)	11 (11.7)	7 (63.6)	8 (53.3)	37 (26.1)
Death	0	0	0	6 (40.0)	6 (4.2)
Transfer of care	1 (4.5)	1 (1.1)	0	1 (6.7)	3 (2.1)
Subspecialty involved					
General surgery	3 (13.6)	52 (55.3)	7 (63.6)	5 (33.3)	67 (47.2)
Ophthalmology	2 (9.1)	0	0	0	2 (1.4)
Otolaryngology	1 (4.5)	1 (1.1)	2 (18.2)	1 (6.7)	5 (3.5)
Urology	7 (31.8)	5 (5.3)	0	2 (13.3)	14 (9.9)
Obstetrics-gynecology	2 (9.1)	23 (24.5)	0	3 (20.0)	27 (19.0)
Orthopedics	5 (22.7)	3 (3.2)	2 (18.2)	4 (26.7)	14 (9.9)
Neurosurgery	2 (9.1)	10 (10.6)	0	0	12 (8.4)

Abbreviation: RFOs, retention of foreign objects.

^a^ Other rare events included surgeons leaving the operating room when patients were unstable (2 of 15 [13.3%]); failure to promptly activate intraoperative transfusion protocols (2 [13.3%]); and inappropriate and unsafe use of medical equipment (such as endoscopic, robotic, or laser instruments) (11 [73.3%]).

Intraoperative burns contributed to 11 never events (8.3%) and included oxygen ignition via electrocautery near the face (7 [63.6%]), electrocautery burn to nonsurgical area (2 [18.2%]), failure to use the manufacturer’s recommendation regarding alcohol-based antiseptic solution (1 [9.1%]), and grounding pad burns (1 [9.1%]). Other rare events included surgeons leaving the operating room when patients were unstable (2 of 15 [13.3%]); failure to promptly activate intraoperative transfusion protocols (2 [13.3%]); and inappropriate and unsafe use of medical equipment (such as endoscopic, robotic, or laser instruments) (11 [73.3%]). Varied subspecialties were subject to never events in our data set (Table 1).

### Analysis of Hospital Characteristics

A total of 559 California hospitals were included in our analysis. Of the 142 surgical error reports, matched hospital data were available in 130 (91.5%). On the basis of procedural volume and never events at those hospitals, the surgical never event rate was 1 per 200 000 operations. Such events took place at 91 hospitals. A single never event was noted at 64 hospitals, whereas 19 hospitals (20.9%) incurred a second penalty, 8 hospitals (8.8%) had 3 citations, 4 hospitals had 3 penalties (4.4%), 4 hospitals had 4 penalties (4.4%), and 2 hospitals had 5 penalties (2.2%). Of the hospitals that had 2 or more penalties, 16 hospitals had additional RFO events. Of the 16 hospitals, 12 (75.0%) had repeat RFO events that occurred in different departments. The rest of the hospitals had multiple penalties that were of different categories (ie, burn, fall, RFO, wrong site or procedure, and product malfunction) (eTable in the Supplement).

An analysis of the incident rates of penalties correcting for surgical volume is summarized in eAppendix 3 in the Supplement. Ultimately, after correcting for surgical volume, no statistically significant associated factors were identified.

### Qualitative Analysis of Improvement Plans

Of the 142 surgical error reports, 129 (90.8%) had legible improvement plans, which were used for subsequent qualitative analysis. The highest coding frequencies and most relevant codes are reported in Table 2. The mean (SD) number of corrective actions per improvement plan was 16 (8) for wrong-site or wrong-patient surgery, 14 (7) for surgical burns, 11 (6) for RFO, and 13 (8) for other. Overall, most represented among action items included policy adherence monitoring (119 [92.2%]), revision of existing policy (84 [65.1%]), and education regarding policy (83 [64.3%]). Disciplinary action toward staff was rare (11 [8.5%]). Policy adherence monitoring includes, per the state, a system by which independent staff directly observe real-time use of safety policies, such as a surgical checklist. Direct observation of competency with new recommended tools or skills was separately recommended in 40 improvement plans (31.0%).

**Table 2.** Corrective Action in Improvement Plans Stratified by Type of Surgical Never Events

Action plan	No. (%) of corrective action plans				
	Total (N = 129)	Wrong procedure/site or patient (n = 20)	RFOs (n = 89)	Surgical burns (n = 11)	Other (n = 9)
Organization and policy					
Policy adherence monitoring	119 (92.2)	20 (100.0)	75 (84.3)	11 (100.0)	9 (100.0)
Revision of existing policies	84 (65.1)	14 (70.0)	55 (61.8)	8 (72.7)	4 (44.4)
Education of revised policy	83 (64.3)	16 (80.0)	51 (57.3)	8 (72.7)	5 (55.6)
Surgical checklist revision or adoption	52 (40.3)	9 (45.0)	52 (58.4)	1 (9.1)	2 (22.2)
Reeducation of current policy	42 (32.6)	9 (45.0)	25 (28.1)	4 (36.4)	3 (33.3)
Disciplinary actions	11 (8.5)	3 (15.0)	NA	1 (9.1)	NA
Verification of surgical site and marking of the site	9 (7.0)	9 (45.0)	NA	NA	NA
Interpersonal communication					
Written communication of instrument counts	42 (32.6)	NA	42 (47.2)	NA	NA
Verbal announcement of completed count	28 (21.7)	NA	28 (31.5)	NA	NA
Verbal announcement of sponge or instrument placement or removal	22 (17.1)	NA	22 (24.7)	NA	NA
Formal teaching to improve communication	17 (13.2)	3 (15.0)	9 (10.1)	2 (18.2)	3 (33.3)
Verbal or audible timeout revised	16 (12.4)	10 (50.0)	4 (4.5)	2 (18.2)	NA
Confirm patient, procedure, or incision site with patient	7 (5.4)	6 (30.0)	NA	NA	1 (11.1)
Tools, technologies, and skills					
Competency validation by direct observation	40 (31.0)	3 (15.0)	28 (31.5)	5 (45.5)	4 (44.4)
Hands-on training	37 (28.7)	1 (5.0)	23 (25.8)	7 (63.6)	5 (55.6)
Procurement of new equipment	32 (24.8)	5 (25.0)	24 (27.0)	3 (27.3)	NA
Passive training (online)	28 (21.7)	1 (5.0)	17 (19.1)	7 (63.6)	3 (33.3)
Setting equipment to safety standards	18 (14.0)	NA	6 (6.7)	6 (54.5)	3 (33.3)

Abbreviations: NA, not applicable; RFOs, retention of foreign objects.

In 59.1% of cases of RFO, revision or adoption of a surgical checklist was recommended. A written record of instrument counts, verbal cues announcing the placement of an object in the body, and verbal announcements for the end of counts were all additional action items. Sixteen hospitals had repeat RFO events, with 12 (75.0%) of these hospitals having repeat RFO events occurring in different departments. Recommendations were not clear if regulator suggestions required hospital-wide adherence or only adherence for the involved team or department. New equipment was only recommended in 32 cases (24.2%). Beyond the use of checklists, novel suggestions from California expert reviewers included using formal US Department of Health and Human Services curriculum, such as TeamSTEPPS (Strategies and Tools to Enhance Performance and Patient Safety Curriculum Training), instituting electronic or radiofrequency object tracking in the operating room, or implementing a visual whiteboard to reduce surgical error (Table 3).

**Table 3.** Specific Examples of Improvements by Category

Category	Examples of corrections
**Organization**	
Revision of existing policies	Revised policies to better reflect WHO Surgical Safety Checklist, Joint Commission National Patient Safety Goals standards, and AORN: Management of the Environment of Care Sponge Count Outside consultation to assess safety culture and provide recommendations for policy revision
Education of revised policy	Education of revised policy via emails to physicians Review policy changes during operating room huddles Formal hands-on training required if new technology or process adopted
Reeducation of current policy	
Policy adherence monitoring	Review of documentation Direct observation of policy or protocol Disciplinary warnings or actions if training not achieved within time frame
Surgical checklist revision or adoption	Revised checklist to better reflect WHO Surgical Safety Checklist Revised checklist to better reflect Joint Commission National Patient Safety Goals standards
Disciplinary actions	Restriction of physician operative privileges Disciplinary warnings and termination if nonadherent
Verification of surgical site and marking of the site	Review of imaging, reports, and preoperative history to verify surgical site Require presentation of imaging in the operating room before incision Team consensus on surgical site before incision
**Interpersonal communication**	
Verbal or audible timeout revised	Require all staff and physicians to pause for timeout Review deficiencies concerning timeout policy Timeout script developed and implemented Nurse looking in medical record, reading through script for each procedure Ensure visibility of surgical site during timeout Verbal confirmation of surgical site by each member of the team
Formal teaching to improve communication	Strategies and Tools to Enhance Performance and Patient Safety curriculum training (ahrq.gov) Situation, Background, Assessment, Recommendation Assertiveness training for staff Emails to surgeons to update policies regarding communication between anesthesia and staff
Confirm patient, procedure, or incision site with patient	Site marked while the patient is in preoperative room Confirm site and procedure with patient and/or family
Verbal announcement of sponge or instrument placement or removal	Decrease ambient noise during count Staff training to improve communication and assertiveness Speak Up for Patient Safety training
Written communication of instrument counts	Visible whiteboard in operating room Intraoperative documentation of instrument counts in electronic record whenever instruments are counted
Verbal announcement of completed count	Pause while final count in progress Final closing count undertaken by scrub tech and nurse before completion of skin closure Surgeon verbally confirms completed count Radiography used if cannot reconcile count Avoid wound packing with towels or sponges
**Tools, technology, and skills**	
Procurement of new equipment	Install racks to hold used sponges to improve organization of sponges and ease of counting Use of radiofrequency sponges Check patient’s body cavity with radiofrequency wand if counts are incorrect Adopt electronic sponge tracking system
Competency validation by direct observation	Assess individual competency, identify individual weaknesses, and allow for immediate corrective action Annual competency reevaluation for surgical counts
Hands-on training	Hands-on training of new count procedures, including use of new instrument counting technologies
Passive training	Lecture using AORN materials Handouts and emails with policy changes
Setting equipment to safety standards	Turn off all open oxygen sources for at least 1 min before using electrical surgical unit or other ignition source. If oxygen cannot be turned off, it should be decreased to minimal possible setting while maintaining patient oxygen saturation. Use nasal cannula instead of face mask when possible. Review xiphoid draping process to prevent trapping of pooled oxygen Confirm power settings before use Keep power at lowest settings

Abbreviations: AORN, Association of Perioperative Registered Nurses; WHO, World Health Organization.

## Discussion

This cross-sectional study found surgical never events at a rate of 1 per 200 000 operations during a decade, representing 142 individual events in California. Hospitals with higher surgical volume were more likely to report never events; however, after correction for surgical volume, no measurable association was found between patient or hospital characteristics and reported events. Repeated events, in particular RFOs, were noted, and as long as the US medical system remains complex, certain errors may be difficult to reduce to zero. Checklist revision, monitoring, and adherence were focal points of criticism in improvement reports. Almost universally, real-time observation of checklist adherence was recommended by state regulators after an incident.

Estimated rates of medical error on a population level are controversial given the difficulty in attributing complications to iatrogenic causes vs the natural history of disease.^23,24^ In this study, such concerns were mitigated given detailed records and multidisciplinary review of each error, a focus on surgery alone, and documentation of consequences. Ultimately, this study’s finding represents a never event rate larger than the previous estimate of approximately 4000 surgical errors annually in 50 million operations across the US.^1^ The frequency of surgical RFOs has been estimated to occur in up to 1 of every 18 000 operations performed and 1 of 1000 abdominal operations.^25^ In this domain, a rate of only 1 in 300 000 operations was found. It is unknown whether this discrepancy is attributable to different reporting mechanisms, true improvements in safety culture, or both. Emergency surgery, unplanned operative changes, and greater patient body habitus may increase the risk of RFOs.^26^ This study’s data lacked the granularity to look at these specific factors.

Novel technologies may specifically reduce the risk of RFOs. Procuring new technology was only suggested as a solution for surgical never events 24.0% of the time in the reviewed improvement reports. Given that sponges, towels, or gauze accounted for 60.6% of RFOs in this data set, available technology could be leveraged to reduce this risk. Radio frequency system–embedded sponges perform better than plain radiography in the detection of retained surgical sponges during emergency surgery.^27^ Computer-aided detection or artificial intelligence may better identify RFOs quickly during intraoperative radiography.^28^ Inexpensive technology, such as incorporating visual cues on empty packages or see-through sponge bag counters, may be economical and beneficial.^29^ Human error itself may be reduced in the far future with robotic technology that complements and potentially supplements human involvement in the operating room.^30^ To what degree these technologies were already used in affected hospitals at the time of the never event is not known.

Despite increasing checklist saturation in the operating room and on the wards, checklists must be used to be effective.^20,31^ Despite California regulators’ focus on checklists in improvement reports, checklists have a mixed track record for safety improvements.^8,9,10,32,33,34^ For example, the adoption of a surgical checklist in Canada was not associated with significant reductions in complications or mortality.^35^ In contrast, a meta-analysis^8^ of 3 studies demonstrated lower pooled risks of death and complications. The difference in findings may be attributable to adherence or the method in which checklists were implemented. To improve adherence, improvement recommendations emphasized real-time observation of checklist use. The Hawthorne effect, or a change in behavior as a response to the attention received through direct observation, would likely increase adherence to checklists. It is unknown whether such tactics ultimately translate to decreased surgical never events.^36^ A major mechanism by which checklists reduce harm may relate to teamwork, communication, and promoting a culture of safety; mandatory initiatives may not lead to improvements in these domains. Nonetheless, the focus on checklists and adherence to checklists in these reports suggests that regulators view this as best practice.

Beyond checklists, deficiencies in technical and nontechnical skills have been implicated as causes of surgical errors.^37^ Therefore, contemporary surgical training must include robust education on communication, team work, leadership, and managing stress, as well as on the technical aspects of surgery itself.^37^ Evidence-based curriculums, such as the TeamSTEPPS, recommended frequently by regulators, is a government-developed, 25-year effort to mitigate medical errors and reduce patient harms. In addition to the moral and ethical duty to prevent never events, these events often result in financial strain to the health care system.^1^ Hospitals should proactively take steps to prevent errors through training, a culture of safety, and open communication. It is hoped that evidence-based study will continue to allow future reduction in surgical errors.

### Limitations

This study has limitations. If not reported by patients or detected during site visits, adverse events were self-reported by each hospital. Hospitals may have varied administrative systems to assuage potential patient concerns and work within the law to limit liability and attention. If present, any never event underreporting may be biased by a variable threshold based on race/ethnicity, patient income, hospital profitability, or hospital culture. Furthermore, events do not include near misses, which would also be of interest.^38^ Given the rarity of events, associations between hospital characteristics and outcomes are more likely to represent chance alone. Documentation of emergency surgery, unplanned operative changes, physician experience, and patient characteristics, such as obesity, may increase the risk of never events, such as RFOs, but data from this study lacked consistent granularity to analyze these specific factors.^26^

## Conclusions

Surgical error remains a complex but rare issue in California. Numerous strategies have evolved to reduce errors, many involving the thorough use of checklists. Real-time observation of adherence to checklists was almost universally recommended after an incident, but further research is needed to prove that such adherence would prevent surgical errors.

## Notes

Supplement. **eAppendix 1.** Coding Index Adapted From the Systems Engineering Initiative for Patient Safety (SEIPS 2.0) Model and the World Health Organization (WHO) Surgical Checklist **eAppendix 2.** Enhancing Transparency in Reporting the Synthesis of Qualitative Research (ENTREQ) **eAppendix 3.** Penalties by Category (Details on Repeated Events in a Single Hospital) **eTable.** Penalties by Category
