# Supplementary material for: Rates of Serious Surgical Errors in California and Plans to Prevent Recurrence
Source: JAMA Netw Open. 2021 May 3;4(5):e217058. doi: 10.1001/jamanetworkopen.2021.7058 (PMC8094010; doi:10.1001/jamanetworkopen.2021.7058)
Supplement: Supplement. — eAppendix 1. Coding Index Adapted From the Systems Engineering Initiative for Patient Safety (SEIPS 2.0) Model and the World Health Organization (WHO) Surgical Checklist eAppendix 2. Enhancing Transparency in Reporting the Synthesis of Qualitative Research (ENTREQ) eAppendix 3. Penalties by Category (Details on Repeated Events in a Single Hospital) eTable. Penalties by Category [file jamanetwopen-e217058-s001.pdf]

Supplementary Online Content

Cohen AJ, Lui H, Zheng M, et al. Rates of serious surgical errors in California and plans to prevent recurrence. *JAMA Netw Open*. 2021;4(4):e217058. doi:10.1001/jamanetworkopen.2021.7058

eAppendix 1. Coding Index Adapted From the Systems Engineering Initiative for Patient Safety (SEIPS 2.0) Model and the World Health Organization (WHO) Surgical Checklist

eAppendix 2. Enhancing Transparency in Reporting the Synthesis of Qualitative Research (ENTREQ)

eAppendix 3. Penalties by Category (Details on Repeated Events in a Single Hospital)

eTable. Penalties by Category

This supplementary material has been provided by the authors to give readers additional information about their work.

© 2021 Cohen AJ et al. *JAMA Network Open.*

eAppendix 1. Coding Index Adapted From the Systems Engineering Initiative for Patient Safety (SEIPS 2.0) Model and the World Health Organization (WHO) Surgical Checklist

**Category** *Subcategory*	**Sub-codes**				
**Interpersonal**					
Education	Training to improve staff OR communication				
Effective communication patterns	Handoff Communication	SBAR	Strong leadership (eg, positive feedback)	Debriefing	
Patient communication	Primary language identified	Translator available			
**Tools, Technology, and Skills**					
Education/Training	Effective training practices (eg, questions encouraged)	Competency validation by direct observation	Simulation		
Elements of strong safety culture	Surgical Site Verification (i.e. patient, family, reports, orders, exams)	Site/Side Marking Requirements	All staff and physicians agree with surgical site	Improved situational awareness	
Equipment	Adequate availability of equipment	Procurement of new equipment			
Privileges	Revision of surgical privileges based on training competencies				
**Organization**					

Review	Internal Administrative Review	Root Cause Analysis (Joint Commission RCA Template)	Formed process improvement team		
Accountability	Disciplinary Warnings	Physician Case Monitoring	OR Staff Instrument monitoring	Annual performance evaluations	
Policy	Revision Preoperative/Procedural Checklist	Joint Commission 2009 NPSG standards	WHO Surgical Safety Checklist	No changes to the policy	
	Revision of Surgical Instrument Checklist	Define countable items	Surgical instrument tracking	Documentation of counts	
	Implementation of Checklist Where There was not a checklist	Radiologically identifiable towels	Verbal announcement of sponge/instrument placement/removal	Do not cut/alter instruments/towels	
	Other				
Monitoring	Audits (Time Out + Site Marking + Checklist)				
Education	Education of Revised Protocol/Policy	Re-education of current policy			
	Verbal/Audible Time-out				
**Internal Environment**					
	Aspects of the intraoperative tasks that enable optimal clinician or team performance (eg, optimized task ergonomics)				

© 2021 Cohen AJ et al. *JAMA Network Open.*

eAppendix 2. Enhancing Transparency in Reporting the Synthesis of Qualitative Research (ENTREQ)

1	**Aim**	To use qualitative methods to summarize regulators' suggestions to hospitals to prevent recurrent events (Introduction paragraph 4)
2	**Synthesis methodology**	A directed qualitative content analysis approach was undertaken (Methods Paragraph 6)
3	**Approach to searching**	Analysis of the text was terminated upon failure to generate additional inductive codes for each improvement plan. (Methods Paragraph 6)
4	**Inclusion criteria**	We defined a surgical never event based on definitions established by publication of the US Department of Health and Human Services National Quality Forum and considered only those that occurred in the operating room setting (Methods Paragraph 3)
5	**Data sources**	CDPH hospital administrative penalty reports (Methods Paragraph 2)
6	**Electronic Search strategy**	The electronic database was accessed. Thereafter all reports were manually reviewed by two authors (MZ & HL) (Methods Paragraph 3)
7	**Study screening methods**	All reports were reviewed by two authors (MZ & HL) for appropriateness for inclusion in the study. For cases when consensus was not achieved a third author (AC) provided arbitration. (Methods Paragraph 3)
8	**Study characteristics**	The content of each improvement report was reviewed in detail, with particular attention to the steps recommended by regulators to each hospital. Additional data collected from each error report included: hospital name, California Office of Statewide Health Planning and Development (OSHPD) hospital identification number, hospital location, the type of never event, subspecialty involved, and the consequence for the patient. (Methods Paragraph 3)
9	**Study selection results**	386 reports screened, 142 reports were ascribable to never events occurring during surgery. (Table 1)
10	**Rationale for appraisal**	A directed qualitative content analysis approach was undertaken based on previously published qualitative research on hospital and surgical risk management. Kolodzey L, et. al. and Hu Y-Y et. al. (Methods Paragraph 7)
11	**Appraisal items**	Use framework of Systems Engineering Initiative for Patient Safety (SEIPS 2.0) model and the World Health Organization (WHO) Surgical Checklist to help characterize and appraise items (Methods Paragraph 6)
12	**Appraisal process**	Double coding ceased once the acceptable degree of intercoder agreement was reached (κ>0.79). The coding scheme was presented to a team of surgeons (AC, BB) who revised the language to more effectively capture surgical practice. (Appendix A) (Methods Paragraph 6)
13	**Appraisal results**	No weight or exclusions were applied to the assessment . The final coding scheme consisted of broad categories (1. Internal Environment, 2. Interpersonal, 3. Organization, and 4. Tools, Technology, and Skills) that were subdivided into 55 sub-codes. (Results in Table 3 and Appendix A)
14	**Data extraction**	This was performed manually. (Methods Paragraph 6)
15	**Software**	MAXQDA (Verbi Software, Berlin) computer-assisted qualitative and mixed methods analysis software (Methods Paragraph 5)
16	**Number of reviewers**	2 (MZ & HL) (Methods Paragraph 4)
17	**Coding**	First a subset of improvement plans were randomly selected and independently double coded. The two coders (HL, BC) then discussed outcomes from the analysis to reach a consensus and collaborated to refine the coding scheme. Intercoder reliability was assessed using Cohen kappa and double coding ceased once the acceptable degree of intercoder agreement was reached (κ>0.79). (Methods Paragraph 6) (Appendix A)

© 2021 Cohen AJ et al. *JAMA Network Open.*

**18**	**Study comparison**	All improvement reports were of the same basic format and content allowing for easy comparisons and collation of codes (Appendix A)
**19**	**Derivation of themes**	Inductive approach, the data drove the creation of themes. (Appendix A)
**20**	**Quotations**	Table 4
**21**	**Synthesis output**	Discussion Section & Results

© 2021 Cohen AJ et al. *JAMA Network Open.*

eAppendix 3. Penalties by Category (Details on Repeated Events in a Single Hospital)

		Penalties (n)	Surgeries (n per 10^6)	Incident Rate	IRR (per 10^6)	95%CI Lower Bound	95%CI Upper Bound	P value
**Teaching Hospital**								
	Yes	12	3.1	3.88	1.00	0.49	1.85	0.96
	No	79	20.5	3.86				
**Metro Setting**[α]	Yes	79	20.8	3.80	0.61	0.28	1.57	0.23
	No	7	1.1	6.20				
**>50% Surgical Population 60 or older**								
	Yes	17	4.6	3.71	0.95	0.53	1.63	0.88
	No	74	19	3.89				
**>50% Surgical Population Medical or Public Aid Insurance**[β]								
	Yes	9	4.0	2.24	0.53	0.24	1.06	0.06
	No	82	19.6	4.19				
**>50% Surgical Population Caucasian**								
	Yes	55	12.8	4.30	1.29	0.83	2.02	0.24
	No	36	10.8	3.30				
[α]Missing Metro Status on 5 penalty reports, [β]County Indigent, Other Indigent, or Medical Insurance Status								

© 2021 Cohen AJ et al. *JAMA Network Open.*

eTable. Penalties by Category

Hospital	Event #1 (Year)	Correction	Event #2 (Year)	Correction	Event #3 (Year)	Correction	Event #4 (Year)	Correction	Event #5 (Year)	Correction
A	**Retained Foreign Object:** A surgical sponge was retained in the pleural cavity after aortic valve replacement and coronary bypasss graft requiring a second procedure. (2009)	- Re-education on surgical counting for all surgical staff<br>- Intraoperative count competency developed and compliance tracked	**Wrong Site/Procedure:** Surgeon started an incision on the wrong side for inguinal orchiectomy. (2011)	- Team STEPPS training<br>- RN and physician to verify site-marking visibility on time out	**Wrong Site/Procedure:** Surgeon removed normal kidney instead of kidney with cancer. (2012)	- imaging to be pulled up before start of operation<br>- The pre-operative safety checklist was revised to include diagnostic image availability verification.	**Fall:** Patient fell from interventional radiology table, requiring an additional surgery for fracture of the 8th thoracic vertebrae. (2012)	- Additional security straps/belts were purchased to accomodate patients with larger body habitus<br>- The imaging manager verify safe patient placement on table	**Retained Foreign Object:** An 18x18 sponge was retained in the patient after cesarean section for 11 weeks. (2014)	- Retrained staff on loading sponge accounting system and counting procedures<br>- Intra-operative counting competency requirement for staff
B	**Wrong Site/Procedure:** Failure to perform time out prior to beginning of surgery led to surgeon proceeding with surgery on the incorrect knee. (2008)	- Staff was re-educated about responsibility to speak up when they believe patient safety is compromised<br>- Monitoring of time out procedures demonstrated full compliance in two consecutive quarters	**Retained Foreign Object:** A retractor blade was retained in the patient after mitral valve repair, requiring a second surgery. (2009)	- OR staff will initiate chain of command if they face resistance from surgical team when complying with surgical count policy<br>- Prior to leaving, the staff will communicate instrument counts to team	**Retained Foreign Object:** A surgical sponge was retained after sigmoid resection and colovesical fistula repair, requiring an additional surgery. (2009)	- Sponge counting revised to use pocketed holding bags<br>- Audible communication between staff after counts are performed	**Retained Foreign Object:** A surgical sponge was retained in a patient after bladder surgery, requiring a second surgery. (2011)	- count policy revised to include process for tracking sponges used for packing<br>- Limit placed on number of sponges on sterile field at the time of count<br>- RN competencies re-evaluated	**Retained Foreign Object:** A drain bulb was not removed from patient's vagina after conclusion of robotic-assisted hysterectomy, leading to admission for infection. The bulb was used to maintain pneumoperitoneum. (2013)	- Surgical counts policy revised to include tracking of all surgical items placed or inserted during procedures<br>- Process developed to track inserted items<br>- OBGYN team identified additional options for maintaining pneumoperitoneum
C	**Product or Device Events:** Non-functioning autoclave led to failure to ensure instruments were completely sterilized prior to surgery. (2007)	None listed	**Fall:** Patient slid off of operating table onto floor with surgical incisions. (2008)	None listed	**Product or Device Events:** Failed to ensure that sterilization staff knew that instruments were supposed to be disassembled before cleaning and processing. (2008)	- Create and maintain employee education log<br>- Create reference photo catalog for new instruments that are undergoing cleaning	**Retained Foreign Object:** Inserted wrong end of lumbar drain to spinal column and upon removal, the tip sheared was retained. Patient readmitted (2009)	- Develop in-service training for placement of lumbar drains<br>- Implement process to cover all new items/kits in the OR		
D	**Retained Foreign Object:** Drill bit was retained in patient's skull	- re-educated on surgical count policy<br>- Staff trained to get an x-ray	**Retained Foreign Object:** A surgical sponge was left in the pelvic cavity after exploratory	- Medical staff re-educated on surgical count policy<br>- Clarified	**Retained Foreign Object:** A plastic clip was left inside a patient's skull after removal of	- Developed a process to count clips during procedures<br>- Revised	**Retained Foreign Object:** A surgical sponge was retained	- Nurses involved in incident were terminated for non-		

© 2021 Cohen AJ et al. *JAMA Network Open.*

	after brain tumor resection. (2008)	whenever a retained foreign body suspected<br>- training to improve communication between OR staff and surgeons	laparatomy The sponge was noted in subsequent CT acquired after the patient continued to have abdominal pain (2010)	circumstances for usage of manual counting of sponges. Sponges should otherwise be counted with a digital scanner.	brain tumor. (2011)	procedures also implemented in the Labor and Delivery and Orthopedic Institute	after exploratory laparotomy with transverse colectomy and tumor debulking. (2011)	compliance<br>- Formal re-training of surgical staff on counting procedures<br>- Ongoing reports to identify count discrepancies		
E	**Medication Error:** Patient given fentanyl patch for acute post-operative pain while getting other opioid medications, leading to hypoxia and code blue, and emergency intubation. (2007)	None listed	**Retained Foreign Object:** A guide wire was retained in the patient's common femoral artery and found 29 days later during a second cardiac catheter procedure. (2009)	- Re-education of the correct process for deployment and removal of angio guide wire for all cardiovascular staff<br>- Audits and in-service performed to ensure competencies met	**Retained Foreign Object:** Large volume of surgical sponges used during exploratory laparotomy for acute bleed which was complicated by retention of surgical sponge, requiring a second procedure for removal. (2009)	- A post-operative radiologic exam will be performed for: 1) a case lasting more than 10 hours, 2) when two or more body cavities are entered, 3) when eight or more units of blood products are given, 4) when baseline count is not completed<br>- No radiopaque items will not be placed on the sterile field after cavity is opened	**Retained Foreign Object:** A temporary pin was retained after spine surgery, causing pain, and requiring a second surgery. (2010)	- Radiologist should read x-ray before patient leaves the OR<br>- Xray if case lasts ≥7 hours, 2) when 3 or more scrubs reliefs occur in the case, 3) an unexpected change in surgical procedure<br>- Include temporary items such as pins and spacers in the surgical count<br>- Re-education on surgical counting		
F	**Retained Foreign Object:** A surgical sponge was retained after removal of an infected peritoneal dialysis catheter and exploration of the abdomen, requiring an additional surgery. (2007)	- The surgeon is to be notified each time count is completed<br>- The surgeon repeats back the count<br>- Education of new procedures to staff and physicians	**Surgery Performed on Wrong Patient:** The hospital mislabeled a prostate biopsy specimen, leading to an unnecessary surgery to remove the prostate and lymph nodes. (2008)	None listed	**Failure to Communicate Critical Labs:** Failure to not type and screen led to delay in transfusion in a patient who lost a large amount of blood after a total knee replacement surgery. (2011)	- an anesthesiology provider must update the pre-operative assessment before treatment or surgery commences, including pre-operative lab tests<br>- Education of new policies to pre-operative staff and the	**Retained Foreign Object:** A retained radiopaque vaginal pack was found in the patient's pelvis after a total vaginal hysterectomy and surgical repair of vaginal wall to correct pelvic organ prolapse, requiring an	-the surgeon will announce the number of sponges packed to the staff<br>- The staff will record the number of sponges packed, even after incision closure<br>- Education of new policy to OR		

© 2021 Cohen AJ et al. *JAMA Network Open.*

						Department of Anesthesiology	additional surgery with bowel resection. (2015)	staff / Obstetrics and Gynecology		
G	**Infection:** Failure to implement a system to identify, report, and investigate surgical site infections for seven patients who underwent cardiopulmonary bypass surgery. (2008)	None listed	**Post-operative Nursing Monitoring:** Nursing failed to assess a patient's deterioration after arriving to surgical floor after hysterectomy and failed to intervene when the patient was showing signs of hypovolemia and bleeding, leading to patient's death the night after surgery. (2008)	- Involved staff were retrained on the clinical criteria used to trigger a Rapid Response<br>- All nursing staff re-educated on use of SBAR for hand-off communication	**Retained Foreign Object:** A retained surgical sponge led to small bowel obstruction, additional hospitalization, and second surgery. (2010)	- Updated count policy to include any object that can be "lost in a patient during surgery"<br>- Educated staff on changes to policy<br>- Improve communication between OR staff and OR leadership on sharing reported incidents				
H	**Retained Foreign Object:** Malleable retractor was retained after an exploratory laparotomy for splenic bleeding, requiring a second surgery. (2008)	- at closing, staff will account for anything that is touching the patient<br>- Whiteboard will reflect any instruments or objects that are touching that patient<br>- Daily staff education at board rounds will include re-emphasis on surgical counting	**Product or Device Events:** Humidity in the OR room was low, creating a risk for a fire during the procedure due to increased risk sparking of surgical equipment. (2009)	- Actions to improve the maintenance and monitoring of humidity in the ORs<br>- If humidity reading is low, staff must immediately report to Plant Operations to initiate work order	**Retained Foreign Object:** A 4x4 gauze was left in the patient after surgical placement of a pacemaker, requiring two additional surgeries and subsequent infection. (2011)	- Revised count policy to include communication between surgeon and staff whenever an instrument/device is placed inside a patient<br>- Use of whiteboard to track any object coming in and out of the surgical field<br>- Re-education of surgical staff on counting policy				
I	**Retained Foreign Object:** A surgical sponge was retained after coronary bypass surgery and was detected on x-ray and CT, requiring a second surgery. (2011)	- OR white boards and clear plastic pocket panels will be used to count all sponges<br>- cases ≤3 hours are to be completed by the same perioperative staff that started the case<br>- Re-education on surgical	**Wrong Site/Procedure:** Failure to confirm marking of site lead to surgery on the wrong spine level for scoliosis and spinal stenosis, requiring a second surgery to correct site. (2010)	- All ORs now have access to so that historical radiology studies can be reviewed before and during the procedure<br>- Re-emphasize and education about proper surgical marking with an emphasis on	**Retained Foreign Object:** A metallic breakaway tab from an implant was retained after spine surgery, requiring second procedure. (2010)	- All breakaway portions of hardware are included in the count<br>- Instrument tray count sheet was changed to reflect new counts<br>- OR staff and sterile processing were educated on counting of breakaway hardware				

© 2021 Cohen AJ et al. *JAMA Network Open.*

		handoff and counting processes		spinal level with all nursing staff and surgeons						
J	**Retained Foreign Object:** A sponge was retained in the abdomen after an exploratory laparotomy for acute abdomen, requiring a second surgery. (2007)	- Implementation of sponge counting bags<br>- Developed a standard dry erase board template for counting<br>- Education of surgical staff on new policy and procedures	**Retained Foreign Object:** A laparotomy pad was retained after an elective cesarean section, leading to post-operative ileus and requiring a second operation to remove the object. (2009)	- Re-education of labor and delivery staff on surgical counting<br>- The supply of 4 x 8 radio-opaque sponges were changed to 4 x 18 radio-opaque sponges with ribbons	**Retained Foreign Object:** A surgical scalp clip was retained in a patient's skull after cranial surgery for a cranial defect, requiring a second surgery. (2013)	- Inclusion of clips in surgical count during neurosurgical procedures<br>- Surgical staff received education on new policies				

© 2021 Cohen AJ et al. *JAMA Network Open.*
